# Dual Influence of Endocannabinoids on Long-Term Potentiation of Synaptic Transmission

**DOI:** 10.3389/fphar.2017.00921

**Published:** 2017-12-19

**Authors:** Armando Silva-Cruz, Mattias Carlström, Joaquim A. Ribeiro, Ana M. Sebastião

**Affiliations:** ^1^Instituto de Farmacologia e Neurociências, Faculdade de Medicina, Universidade de Lisboa, Lisbon, Portugal; ^2^Instituto de Medicina Molecular, Faculdade de Medicina, Universidade de Lisboa, Lisbon, Portugal; ^3^Department of Physiology and Pharmacology, Karolinska Institutet, Stockholm, Sweden

**Keywords:** endocannabinoids, cannabinoid CB_1_ receptor, long-term potentiation, adenosine A_1_ receptor, hippocampus

## Abstract

Cannabinoid receptor 1 (CB_1_R) is widely distributed in the central nervous system, in excitatory and inhibitory neurons, and in astrocytes. CB_1_R agonists impair cognition and prevent long-term potentiation (LTP) of synaptic transmission, but the influence of endogenously formed cannabinoids (eCBs) on hippocampal LTP remains ambiguous. Based on the knowledge that eCBs are released upon high frequency neuronal firing, we hypothesized that the influence of eCBs upon LTP could change according to the paradigm of LTP induction. We thus tested the influence of eCBs on hippocampal LTP using two θ-burst protocols that induce either a weak or a strong LTP. LTP induced by a weak-θ-burst protocol is facilitated while preventing the endogenous activation of CB_1_Rs. In contrast, the same procedures lead to inhibition of LTP induced by the strong-θ-burst protocol, suggestive of a facilitatory action of eCBs upon strong LTP. Accordingly, an inhibitor of the metabolism of the predominant eCB in the hippocampus, 2-arachidonoyl-glycerol (2-AG), facilitates strong LTP. The facilitatory action of endogenous CB_1_R activation does not require the activity of inhibitory A1 adenosine receptors, is not affected by inhibition of astrocytic metabolism, but involves inhibitory GABAergic transmission. The continuous activation of CB_1_Rs via exogenous cannabinoids, or by drugs known to prevent metabolism of the non-prevalent hippocampal eCB, anandamide, inhibited LTP. We conclude that endogenous activation of CB_1_Rs by physiologically formed eCBs exerts a fine-tune homeostatic control of LTP in the hippocampus, acting as a high-pass filter, therefore likely reducing the signal-to-noise ratio of synaptic strengthening.

## Introduction

The influence of marijuana upon human cognition mostly results from its ability to interfere with the action of endocannabinoids (eCBs) in the brain. eCBs are widely recognized as fine-tune modulators of synaptic activity, their action mainly resulting from activation of G protein-coupled cannabinoid receptor type 1 receptors (CB_1_R), which are widely distributed in the central nervous system, in particular in the hippocampus, cortex, basal ganglia, and cerebellum (Herkenham et al., 1991; Matsuda et al., 1993; Tsou et al., 1998; Marsicano and Lutz, 1999; Wilson and Nicoll, 2002). CB_1_Rs are localized in neurons, both excitatory and inhibitory (Katona et al., 2001; Wilson et al., 2001; Kawamura, 2006; Hoffman et al., 2010), and also in astrocytes (Navarrete and Araque, 2008). CB_1_Rs are endogenously activated by eCBs, mainly the fatty acid derivatives 2-arachidonoyl-sn-glycerol (2-AG) and anandamide. eCB synthesis mostly results from cleavage of postsynaptic membrane lipids as a consequence of the activation of postsynaptic G-coupled glutamate metabotropic receptors, which are predominantly activated as a consequence of high rate of neuronal firing (Chevaleyre et al., 2006; Katona et al., 2006). eCBs thus travel in a retrograde manner to activate astrocytic and nerve-terminal located CB_1_R, resulting in inhibition of neurotransmitter release, and giving rise to several forms of short-term synaptic plasticity (Freund et al., 2003; Chevaleyre et al., 2006; Kano et al., 2009; Ohno-Shosaku et al., 2012). While the inhibitory action of eCBs upon neurotransmitter release is quite consistent, their action upon synaptic plasticity induced by brief high frequency neuronal firing, as long-term potentiation (LTP), is much more controversial. Indeed, and considering only the hippocampus, a brain area important for memory encoding and the mostly used to study synaptic plasticity phenomena, there are reports showing that eCBs restrict LTP (Bohme et al., 1999; Slanina et al., 2005) while others show that they facilitate LTP (Carlson et al., 2002; De Oliveira Alvares et al., 2006). This is intriguing since LTP is a compelling cellular model for learning and memory (see Nicoll, 2017), and exogenous cannabinoids, including the phytocannabinoids present in marijuana and the synthetic CB_1_Rs agonists, have a negative impact upon learning and memory in humans and in laboratory animals (Miller et al., 1977; Lane et al., 2005; Sousa et al., 2011; Mouro et al., 2017). Elegant studies aiming at understanding the influence of eCBs upon LTP in different cell types or circuits in the hippocampus show that the action of eCBs may vary according to the cell type where the CB_1_Rs sit (Monory et al., 2015) as well as the hippocampal circuit where LTP is induced (Wang et al., 2016). Knowing that eCBs are formed as a function of neuronal activity, we hypothesized that the influence of eCBs upon LTP could also vary as a function of the pattern of neuronal firing that induces plasticity. Evidence for that would not only contribute to further clarify reasons for discrepant data in the literature but also to better insight on the subtleties eCBs use to control synaptic strengthening. The present work was thus designed to evaluate the influence of eCBs upon hippocampal LTP induced by two types of stimulation, while keeping a θ-burst stimulation pattern, known to be related to hippocampal-dependent memory function (Buzsáki, 2002). We used a weak or a strong-θ-burst train of stimulation since previous evidence lead us to hypothesize that modulation of strong or weak forms of LTP may differ. Data obtained allow to suggest that eCBs act as a high pass filter, inhibiting LTP of low magnitude while facilitating robust LTP. Thus, eCBs likely reduce the signal-to-noise ratio of activity-dependent synaptic strengthening at the CA1 area of the hippocampus.

## Materials and Methods

### Animals

The experimental protocols were approved by Institutional Animal Care and Use Committee (IACUC) from Stockholm (Sweden) or Lisbon (Portugal), and conducted in accordance with Portuguese and Swedish legislation on animal care and the European Community guidelines (Directive 2010/63/EU).

Most of the experiments were performed using male C57Bl6/J mice, aged between 8 and 18 weeks (most frequently 9–13 weeks) (Charles River Laboratories, Paris). In some cases, male and female mice were used to maximize the use of A1R knockout mice; because of this, control experiments using male and female mice have been performed. No appreciable differences between data obtained in males or females were detected (**Supplementary Figure S1**). The adenosine A_1_ receptor knockout (A_1_R^-/-^) and wild-type (A_1_R^+/+^) mice were generated by inactivating the second protein coding exon of the mouse A_1_R gene, from heterozygous breeding pairs with C57Bl6/J background strain (Johansson et al., 2001), obtained from a breeding colony derived from this original line that is housed at Karolinska Institutet, Sweden, and genotyped as described previously (Yang et al., 2015). All animals were social housed under standardized conditions of light (12-h light/12-h dark cycle), temperature (22–24°C), humidity (55–65%), and environmental enrichment (cardboard tubes plus nest material) and had free access to food and tap water.

### Hippocampal Slices

Hippocampal slices were prepared as previously (e.g., Diógenes et al., 2004). The animals were sacrificed by decapitation under deep isoflurane anesthesia. The hippocampus was dissected free within ice-cold artificial cerebrospinal fluid (aCSF) solution composed of (millimeter): NaCl 124, KCl 3, NaHCO_3_ 26, Na_2_HPO_4_ 1.25, MgSO_4_ 1, CaCl_2_ 2; and glucose 10, previously gassed with 95% O_2_ and 5% CO_2_, pH 7.4. Slices (400-μm thick) were cut perpendicularly to the long axis of hippocampus with a McIlwain tissue chopper and allowed to recover functionally and energetically for 1 h in a resting chamber filled with the same solution, at room temperature and continuously gassed.

### Extracellular Recordings

For electrophysiological recordings of field excitatory post-synaptic potentials (fEPSP), individual slices were transferred into a submerged recording chamber (dual submerged chamber) over the nylon mesh and continually superfused with gassed aCSF solution at a constant flow (3 ml/min) and temperature (32°C). This allows oxygenation in both slice surfaces while permitting a relatively fast flow rate to facilitate drug replacement. Stimulation (rectangular 0.1 ms pulses, once every 20 s) was delivered through a concentric electrode placed on Schaffer collateral-commissural fibers, in the stratum radiatum near the CA3–CA1 border. The intensity of the stimulus was set to the one eliciting near 50% of the maximal response, and was maintained throughout the experiment except in those experiments designed to perform input–output curves. In such experiments, after a stabilization period under the standard stimulation conditions, the stimulus intensity was increased by 20 μA every 6 min, within a range of 80–300 μA. fEPSP recording was through a microelectrode filled with NaCl 4 M (2–6 MΩ resistance), placed in CA1 stratum radiatum, coupled to an Axoclamp 2B Amplifier (Axon Instruments) and digitized BNC-2110 (National Instruments). Individual responses were monitored, and averages of six consecutive responses were continuously stored on a personal computer with the WinLTP Software (Anderson and Collingridge, 2001). fEPSPs were continuously recorded under basal stimulation frequencies and LTP was induced only after obtaining stable fEPSP slope values for at least a 15 min. Test drugs were added to the perfusing aCSF at least 30 min before LTP induction, or initiation of the input–output curves. Changes in stimulus frequency (LTP induction) or intensity (input–output curves) were only initiated after at least a 15 min stable baseline in the presence of the drugs.

Long-term potentiation was induced by θ-burst stimulation. Two different stimulation paradigms were used in different experiments, weak-θ-burst and strong-θ-burst protocols, which differed only in the number of trains delivered. The weak-θ-burst consisted of five trains whereas the strong-θ-burst was composed of 10 trains, in both cases the stimulation trains were separated by 200 ms. In both paradigms each train was composed of four stimuli delivered at 100 Hz. LTP magnitude was quantified as the % change in the average fEPSP slopes recorded from 50 to 60 min after LTP induction, taking as 0% the averaged fEPSP slope recorded for 10 min immediately before LTP induction. Throughout the text, while referring to weak LTP or to strong LTP we mean LTP induced by a weak-θ-burst or by a strong-θ-burst, respectively.

### Drugs

The following drugs were used: WIN55,212-2 (WIN) mesylate, AM251, 1,3-dipropyl-8-cyclopentyl-xanthine (DPCPX), picro toxin (PTX), JZL 184, and JZL 195 from Tocris. SR141716A (Rimonabant), tetrahydrolipstatin (Orlistat) from Biogen, URB597 from Cayman Chemicals, barium salt of DL-fluorocitric acid from Sigma-Aldrich.

WIN55,212-2 was used as CB_1_R agonist at a concentration (500 nM) 250 times higher than its *K*_i_ value for these receptors (Kuster et al., 1993). AM251 was used as a CB_1_ receptor inverse agonist at a concentration (1 μM) 100 times higher than its *K*_i_ value for these receptors (Lan et al., 1999). Rimonabant was used as a CB_1_ receptor antagonist at a concentration (1 μM) 500 times higher than its *K*_i_ for this receptor (Rinaldi-Carmona et al., 1994). DPCPX was used as an adenosine A_1_ receptor antagonist at a concentration (50 nM) 100 times higher than its *K*_i_ value for this receptor (Bruns et al., 1987). PTX was used as a GABA_A_ receptor antagonist at a concentration (50 μM) 100 times higher than its *K*_i_ value this receptor (Mehta and Ticku, 2013). JZL 184 was used as potent and selective monoacylglycerol lipase (MAGL) inhibitor at concentration (1 μM) 125 times higher than its IC_50_ for this enzyme (Long et al., 2009a). JZL 195 was used as potent inhibitor of both fatty acid amide hydrolase (FAAH) and of MAGL at a concentration (1 μM), respectively, 500 and 250 times higher than its IC_50_ for these enzymes (Long et al., 2009b). Orlistat was used as a diacylglycerol (DAG) lipase inhibitor at a concentration (10 μM) 100 times higher than the IC_50_ to inhibit DAG lipases α (Bisogno et al., 2006). URB597 was used as a selective FAAH inhibitor at a concentration (1 μM) 200 times the IC_50_ to inhibit this enzyme (Kathuria et al., 2003). Care was taken to use drug concentrations within selectivity ranges and according to previously published work using the same drugs for similar purposes. Solutions of all these drugs were prepared as stock solutions in 100% dimethylsulfoxide (DMSO). Aliquots of these stock solutions were diluted in aCSF in the day of the experiment. The concentration of the stock solution was chosen so that the final concentration of DMSO in the perfusion solutions was ≤0.1% (v/v).

Sodium fluorocitrate, an astrocyte metabolism inhibitor (Bonansco et al., 2011), was prepared as described by Paulsen et al. (1987): 8 mg of the barium salt of DL-fluorocitric acid was dissolved in 0.1 M HCl, precipitated by the addition of 0.1 M Na_2_SO_4_, buffered with 0.1 mM Na_2_HPO_4_ and centrifuged at 1000 × *g* for 5 min; the supernatant containing fluorocitrate was added to aCSF at a final concentration of 200 μM (pH 7.4).

### Statistical Analysis

Data are expressed as the mean ± SEM; *n* corresponds to the number of experiments; in each experiment, only one slice was used per drug condition. At least one drug condition and the corresponding control was tested in each experimental day. Statistical significance was assessed by two-tailed Student’s *t*-test when comparing two groups, or by one-way ANOVA with treatment as the between-subject factor, followed by Sidak’s *post hoc* test when comparing multiple experimental groups. For the input–output curves, statistical significance was assessed by two-way ANOVA with treatment as the between-subject factor, followed by Sidak’s *post hoc* test when comparing multiple experimental groups. A *p*-value of <0.05 was considered to account for significant difference. Analyses were performed with the GraphPad Prism 6 Software.

## Results

### Physiologically Released Endocannabinoids Reduce LTP Induced by a Weak-θ-Burst

The first series of experiments was designed to evaluate the influence of eCBs upon weakly induced LTP. The influence of eCBs was assessed by testing the consequences of drugs that prevent CB_1_R activation by eCBs or the synthesis of eCBs. We focused upon the synthesis of a predominant eCB at the hippocampus, 2-AG (Piyanova et al., 2015).

**FIGURE 1:**
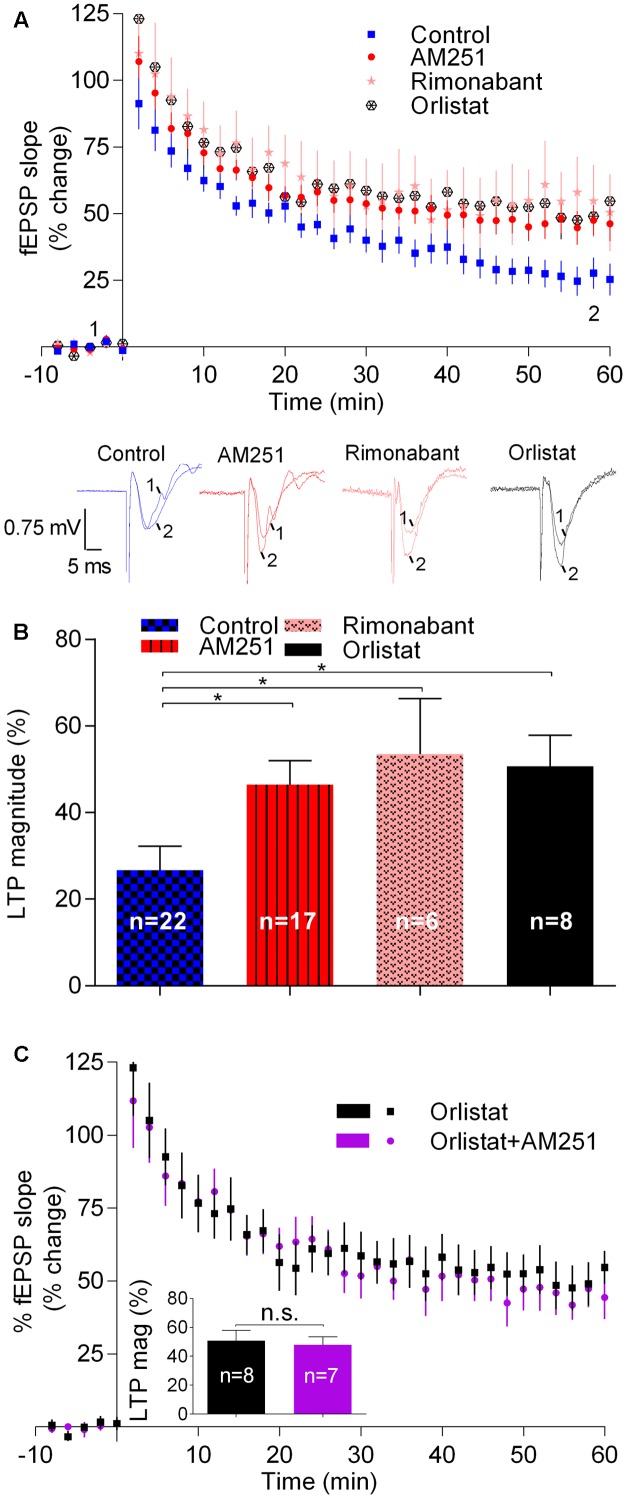
Endocannabinoids inhibit LTP induced by weak-θ-burst stimulation (five trains of 100 Hz, 4 stimuli, separated by 200 ms). **(A)** Time course of the averaged fEPSP slopes in control conditions (no drugs) or in the presence of 1 μM AM251 (CB_1_R inverse agonist), 1 μM Rimonabant (CB_1_R antagonist), or 10 μM Orlistat (a fatty acid synthesis inhibitor). Data are represented as % of the averaged fEPSP slope recorded for 10 min before LTP induction, which were taken as zero%. Original traces taken from representative individual experiments and recorded during the baseline (1) and 50–60 min after weak-θ-burst induction (2) are shown below the time course panel. Each trace is composed by the stimulus artifact, followed by the presynaptic volley and the fEPSP. **(B)** Quantification of LTP magnitude under the indicated drug conditions. LTP magnitude was quantified as the % increase in fEPSP slope recorded at the 50–60 min after LTP induction, compared to the value recorded during the 10 min immediately before LTP induction; zero% represents no LTP and 100% would correspond to fEPSP slopes (at 50–60 min after LTP induction) twice the value recorded before LTP induction. ^∗^*p* < 0.05 (*F*_(4,51)_ = 2.986, one-way ANOVA with Sidak’s correction). **(C)** Non-additivity of the facilitatory action of AM251 and Orlistat, when added together. Data are represented as time course of fEPSP slopes and inset shows average LTP magnitude (LTP mag, defined as in **B**) in the two conditions, the color of the bars corresponding to the color of the symbols in the time course. Data for Orlistat in **(A)** and **(C)** (time course) and in **(B)** and **(C)** (LTP magnitude) are repeated to allow comparison between the action of Orlistat in the absence or presence of AM251. All values are mean ± standard error of mean (SEM) from *n* experiments; *n* values are indicated on the bars. *F*_(7,6)_ = 1.8, ns: *p* > 0.05 (Student’s *t*-test).

In control slices, fEPSP slopes recorded 50–60 min after inducing LTP with a weak-θ-burst, were 26.7 ± 5.5% higher than before LTP induction (*n* = 18; **Figure 1**). In slices where the CB_1_R inverse agonist, AM251 (1 μM) was added to the perfusion at least 30 min before LTP induction, the magnitude of LTP was 46.5 ± 5.4% (*n* = 17, *t* = 2.6, *p* < 0.05 vs. control, **Figure 1**), which corresponds to near 80% increase in LTP magnitude. A similar result was obtained in the presence of another CB_1_R blocker, the selective CB_1_R antagonist, rimonabant (1 μM) (LTP magnitude: 53.5 ± 12.8%, *n* = 6, *t* = 2.5, *p* < 0.05 vs. control, **Figure 1**). In the presence of Orlistat (10 μM), an inhibitor of DAG lipase, the enzyme responsible for the conversion of DAG into 2-AG, the magnitude of LTP was also enhanced toward 50.7 ± 7.2% (*n* = 8, *t* = 2.5, *p* < 0.05; **Figure 1**). Importantly, when both CB_1_R activation and 2-AG synthesis were prevented together, by the simultaneous presence of AM251 (1 μM) and Orlistat (10 μM) the magnitude of LTP was enhanced at the same degree as obtained with each of the drugs alone (*t* = 0.3, *p* > 0.05, **Figure 1C**). This lack of additivity indicates that both drugs facilitate LTP due to their common ability to prevent eCB signaling.

Summarizing, the above results show that drugs known to prevent the activation of CB_1_R by eCBs or drugs known to inhibit the synthesis of 2-AG, the predominant eCB in the hippocampus (Piyanova et al., 2015), cause a marked facilitation of LTP induced by a weak-θ-burst, thus suggesting that eCBs inhibit such form of LTP.

### Physiologically Released Endocannabinoids Enhance LTP with a Strong-θ-Burst

**FIGURE 2:**
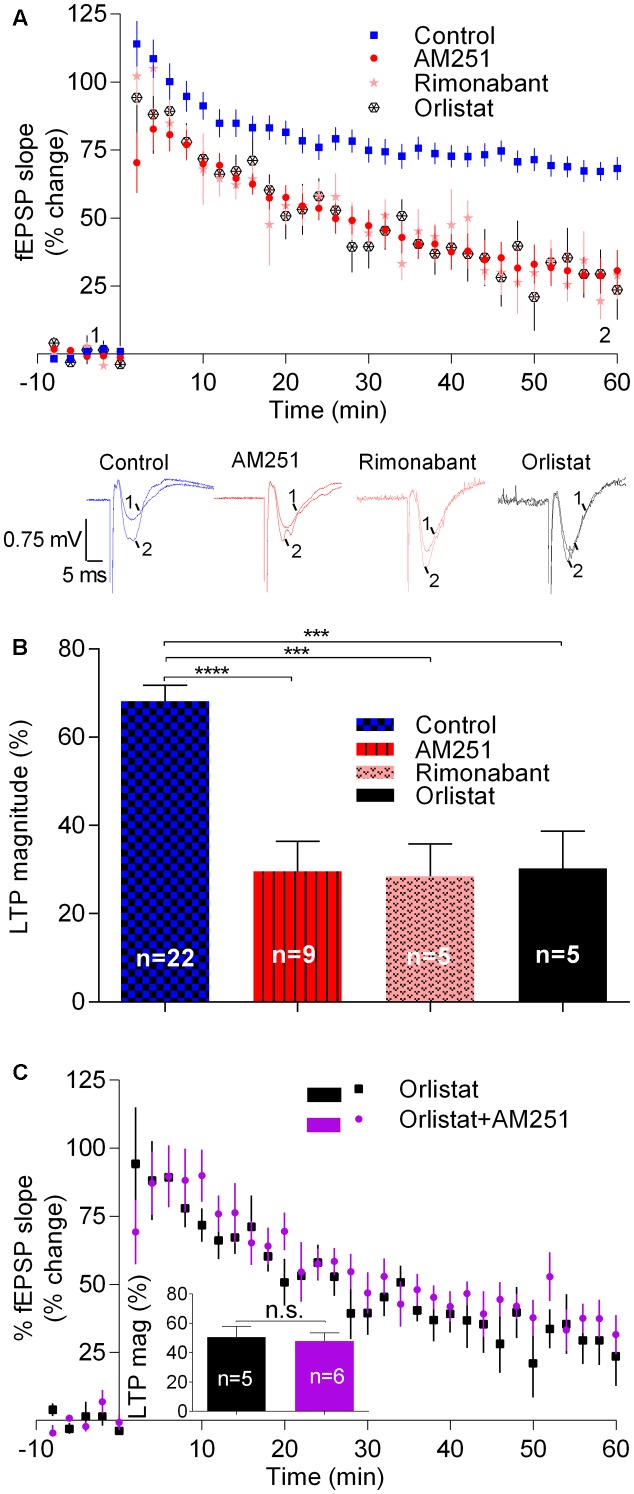
Endocannabinoids enhance LTP induced by strong-θ-burst stimulation (10 trains of 100 Hz, 4 stimuli, separated by 200 ms). **(A)** Time course of the averaged fEPSP slopes, and original traces of fEPSP recordings, in control conditions (no drugs) or in the presence of 1 μM AM251 (CB_1_R inverse agonist), 1 μM Rimonabant (CB_1_R antagonist), or 10 μM Orlistat (a fatty acid synthesis inhibitor). **(B)** Quantification of LTP magnitude under the indicated drug conditions. ^∗∗^*p* < 0.01; ^∗∗∗^*p* < 0.001 (*F*_(7,91)_ = 11.0, one-way ANOVA with Sidak’s correction). **(C)** Non-additivity of the inhibitory effect of AM251 and Orlistat, when added together. ns: *p* > 0.05 (*F*_(4,5)_ = 1.4, Student’s *t*-test). Data for Orlistat in **(A)** and **(C)** (time course) and in **(B)** and **(C)** (LTP magnitude) are repeated to allow comparison between the action of Orlistat in the absence or presence of AM251. For further details see legend to **Figure 1**.

We then assessed the influence of eCBs on LTP induced by a strong-θ-burst protocol, all other experimental conditions being similar to those used before. Fifty–sixty minutes after the strong-θ-burst stimulation, LTP magnitude in control conditions was 68.1 ± 3.7% (*n* = 22) of pre-θ-burst stimulation. LTP dropped off by around 40% in the presence of AM251 (29.6 ± 6.8%, *n* = 9, *t* = 5.1, *p* < 0.001; **Figure 2**) or of rimonabant (28.5 ± 7.4%, *n* = 5, *t* = 4.2, *p* < 0.01; **Figure 2**). In the presence of Orlistat, the magnitude of LTP also decreased toward similar values (30.3 ± 8.4%, *n* = 5, *t* = 4.0, *p* < 0.01; **Figure 2**). It is worthwhile to note that in what concerns to the inhibition of LTP induced by a strong-θ-burst, the effect of AM251 was also not additive with that of Orlistat. Indeed, when both drugs were present, the LTP magnitude was 38.5 ± 6.4% (*n* = 6), a value significantly different (*t* = 0.8, *p* < 0.05) from that obtained in control conditions, but of similar magnitude as that obtained in the presence of each of the drugs separately (**Figure 2**). Again, this suggests that the ability of these drugs to inhibit strongly induced LTP results from their common ability to prevent eCB signaling.

Summarizing, the results reported in this section show that drugs known to prevent CB_1_R activation by eCBs or to inhibit 2-AG synthesis lead to an inhibition of LTP induced by strong-θ-burst. These data are in clear contrast with what was observed when inducing LTP with a weak-θ-burst, and suggest that LTP induced by a strong-θ-burst is facilitated by eCBs.

### Continuous Stimulation of CB_1_R or the Non-prevalent eCB Leads to LTP Inhibition

The approach described in the previous sections was always directed toward the consequences of preventing CB_1_R activation by eCBs. On the light of what is known about the inhibitory action of cannabinoids on neuronal activity, our finding that LTP induced by a strong-θ-burst is reduced by preventing CB_1_R activation was unexpected. We thus decided to assess how this form of LTP is affected by continuous activation of CB_1_Rs. To do so we used two approaches: (1) test the influence of inhibitors of eCB hydrolysis and in such way create conditions for sustained enhanced levels of eCBs, or to (2) use a CB_1_ receptor agonist to exogenously activate CB_1_Rs in a sustained way.

**FIGURE 3:**
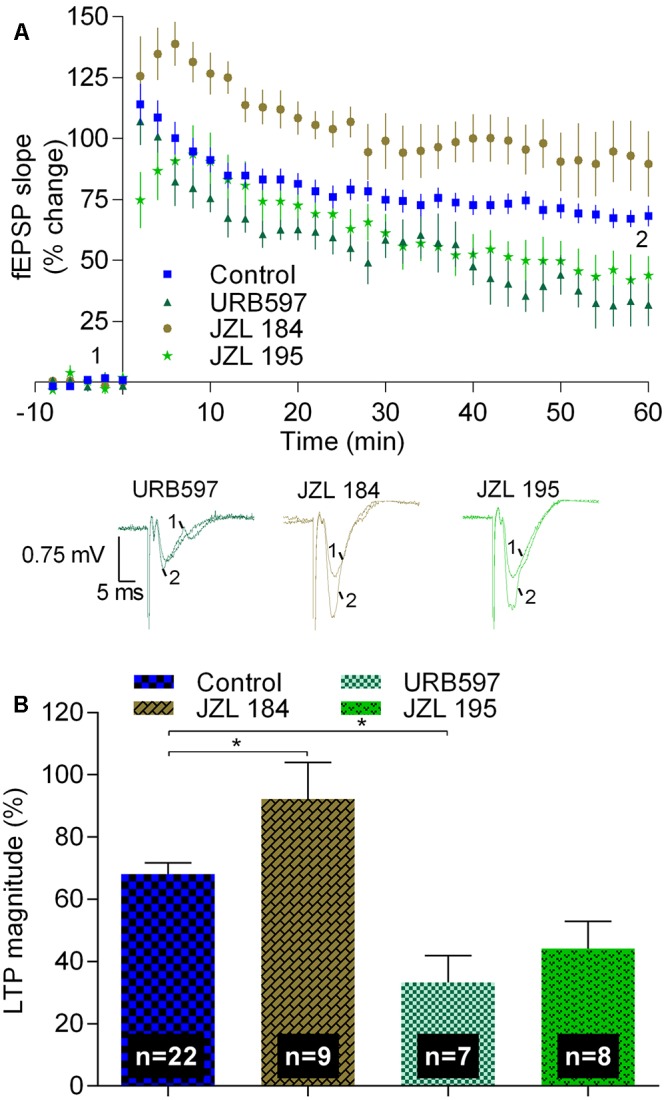
Inhibitors of the hydrolysis of 2-AG or of anandamide differently affect strong-θ-burst induced LTP. **(A)** Time course of the averaged fEPSP slopes, and original traces of fEPSP recordings, in control conditions (no drugs) or in the presence of 1 μM JZL 184 (inhibitor of the enzyme that hydrolyses 2-AG, MAGL), 1 μM URB597 (inhibitor of the enzyme that hydrolyses anandamide, FAAH), or in the presence of 1 μM JZL 195 (inhibitor of FAAH and MAGL). **(B)** Quantification of LTP magnitude under the indicated drug conditions. Data in control conditions, in panels **(A)** and **(B)**, are the same as shown in **Figures 2A,B**, but are represented in this figure to allow comparison with the drug conditions. ^∗^*p* < 0.05 (*F*_(3,42)_ = 10.3, one-way ANOVA with Sidak’s correction). For further details see legend to **Figure 1**.

Data shown in **Figure 3** summarize the findings while using inhibitors of enzymes that prevent hydrolysis of eCBs. When using JZL 184 (1 μM), a selective inhibitor of MAGL, the enzyme that hydrolyses 2-AG, the magnitude of LTP was enhanced toward 92.3 ± 11.2% (*n* = 9, *t* = 2.6, *p* < 0.05 as compared with absence of drugs, **Figure 3**), corresponding to a value about 40% higher than that obtained in the absence of any drug. This finding suggests that enhancement of the levels of the predominant eCB in the hippocampus, 2-AG (Piyanova et al., 2015), facilitates strong LTP, thus in line with previous results showing that blockade of CB_1_R or inhibition of synthesis of 2-AG inhibit strong LTP.

Remarkably, in the presence of URB 597, which at the concentration used (1 μM) inhibits FAAH, but not MAGL, the magnitude of LTP decreased toward 33.3 ± 8.6% (*n* = 7, *t* = 3.4, *p* < 0.05 as compared with absence of drugs, **Figure 3**), thus toward near half of the value obtained in control conditions. Since FAAH hydrolyses anandamide, this data suggest that accumulation of the non-predominant eCB in the hippocampus, anandamide (Piyanova et al., 2015), inhibits LTP in clear contrast with what occurs with the influence of the most abundant eCB in the hippocampus, 2-AG. This conclusion is further supported by the experiments where a non-selective inhibitor of both enzymes, FAAH and MAGL, was used. Thus, in the presence of JZL 195 (1 μM), the LTP magnitude was decreased toward a value (44.2 ± 8.8%, *n* = 8, **Figure 3**) between that obtained with URB 597 and that obtained in the absence of any drug, being not significant different (*t* = 2.4, *p* > 0.05) from any of these conditions. Altogether, the data with JZL 184, URB 597, and JZL 195 allow to suggest that enhanced production of 2-AG and enhanced production of anandamide affect strong LTP in an opposed way. However, the possibility that the inhibitory action of URB 597 results from non-CB1-related mechanisms (Kathuria et al., 2003; Ratano et al., 2017) cannot be fully excluded.

**FIGURE 4:**
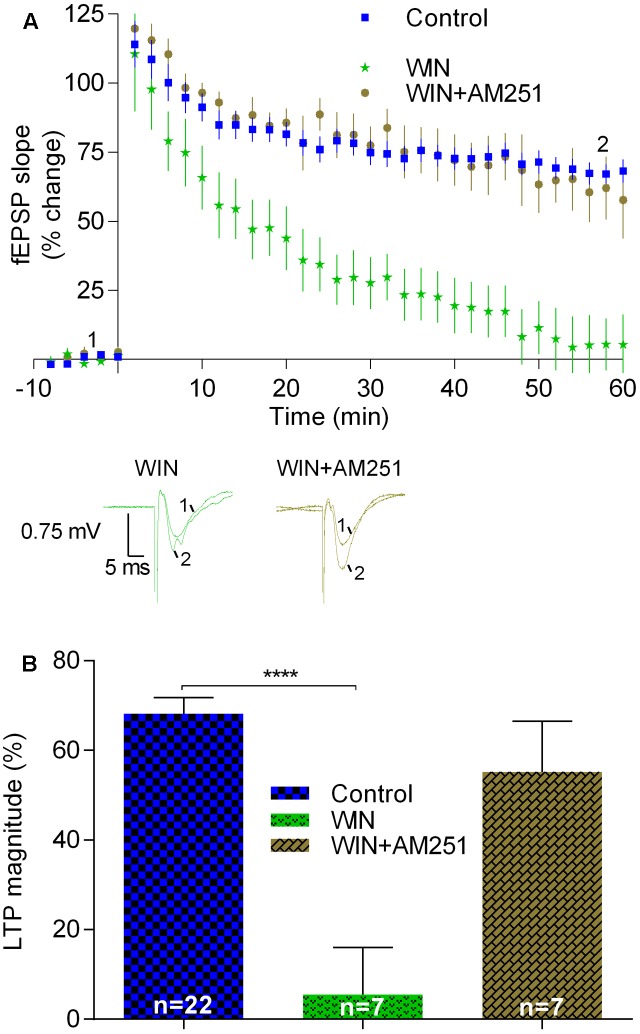
LTP induced by strong-θ-burst is inhibited when CB_1_Rs are continuously activated. **(A)** Time course of the averaged fEPSP slopes, and original traces of fEPSP recordings, in control conditions (no drugs) or in the presence of 500 nM WIN (CB_1_R agonist), or 500 nM WIN together with 1 μM AM251. **(B)** Quantification of LTP magnitude under the indicated drug conditions. Data in control conditions in panels **(A)** and **(B)** are the same as shown in **Figures 2A,B**, but is represented in this figure to allow comparison with the drug conditions. ^∗∗∗∗^*p* < 0.0001 (*F*_(2,33)_ = 10.3, one-way ANOVA with Sidak’s correction). For further details see legend to **Figure 1**.

Secondly, we tested the effect of WIN (500 nM), a compound known to activate CB_1_R. Since the effect of WIN upon synaptic transmission is known to be rather slow (Serpa et al., 2009), the slices were pre-incubated with WIN for at least 60 min before transfer to the acquisition chamber. Then, the slices were stabilized for at least 20 min, LTP being only induced when fEPSP slope values remained stable for at least 15 min. In such experiments LTP was virtually abolished (LTP magnitude: 5.5 ± 10.5%, *n* = 7, *t* = 6.5, *p* < 0.05 vs. pre-LTP induction; *p* < 0.0001 vs. LTP magnitude in control conditions; **Figure 4**). This inhibitory effect of WIN was prevented when the slices had been pre-incubated with the CB_1_R inverse agonist, AM251, before addition of WIN. Indeed, under such conditions the inhibitory effects of both the agonist and the antagonists seem to be reciprocally canceled since LTP magnitude obtained in slices in the presence of AM251 and WIN (55.2 ± 11.2%, *n* = 7, **Figure 4**) was similar (*t* = 1.3, *p* > 0.05) to that obtained in the absence of any drug.

Summarizing, the data reported in this section suggest that sustained activation of CB_1_Rs induced by adding an exogenous agonist as well as prevention of degradation of the non-prevalent eCB in the hippocampus leads to inhibition of LTP induced by the strong-θ-burst. This is in clear contrast with the conclusions that could be drawn while assessing the action of a drug known to prevent the hydrolysis or prevent the formation of the predominant eCB in the hippocampus as well as when accessing the action of transiently released eCBs by using CB_1_R blockers. Altogether, the data indicate that while physiologically released eCBs are required to facilitate LTP induced by a strong θ-burst, non-physiological activation of CB_1_R leads to inhibition of this form of LTP.

### Inhibition of LTP during CB_1_R Blockade Is Not a Result of Enhanced A_1_R Activation

The above results indicating that physiologically released eCBs can facilitate LTP lead us to hypothesize that the strong-θ-burst could lead to the recruitment of other neuromodulators that would affect the neuromodulatory influence of eCBs. Purines are released during high-frequency neuronal firing (Cunha et al., 1996) and adenosine is known to inhibit LTP through activation of A_1_R (De Mendonça and Ribeiro, 2000; Wang et al., 2016), which are abundantly expressed in the hippocampus. Furthermore, A_1_R can affect CB_1_R signaling (Hoffman et al., 2010; Sousa et al., 2011). To test if the apparent facilitatory action of eCBs upon strong-θ-burst-induced LTP could be due to any interference with endogenous adenosine acting on A_1_R, we used two different approaches: genetic (A_1_R^-/-^ mice) or pharmacological (selective A_1_R antagonist, DPCPX) prevention of A_1_R activity.

**FIGURE 5:**
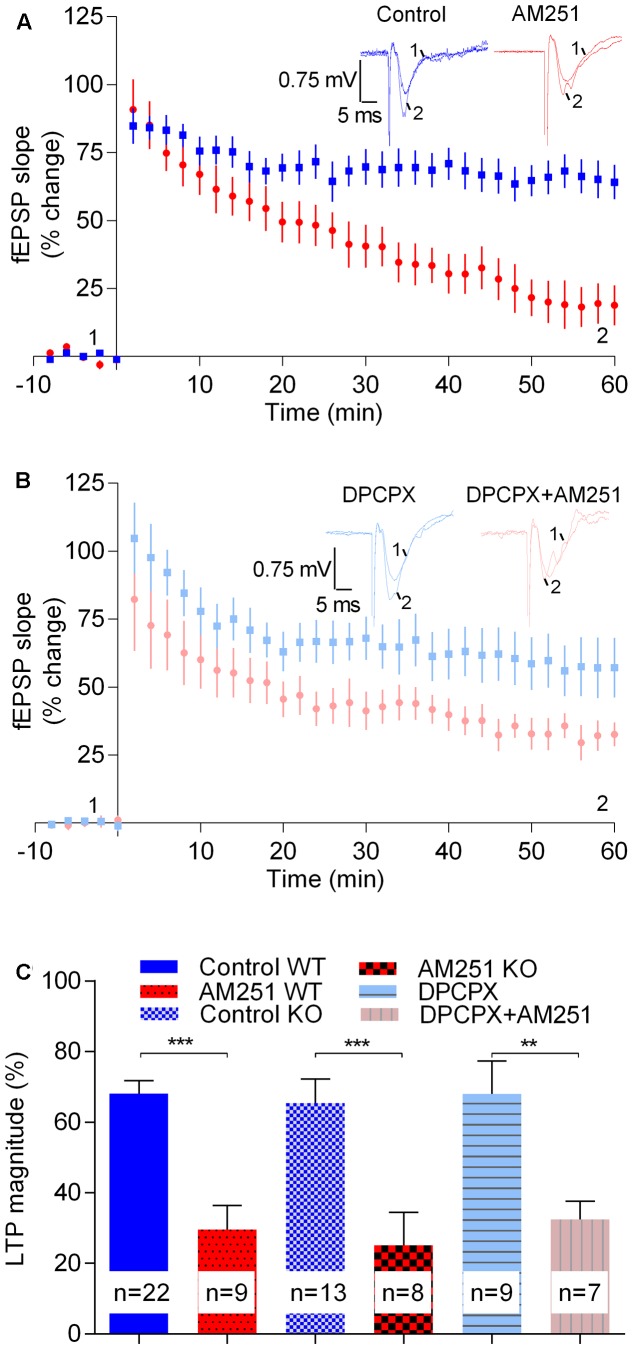
The enhancement LTP caused by physiologically released eCBs does not result from enhanced adenosinergic tonus on A_1_R. **(A)** Data obtained in slices taken from A_1_R KO mice, LTP being induced by a strong-θ-burst in control conditions (no drugs, blue symbols and traces) or in the presence of 1 μM of AM251 (red symbols and traces). **(B)** Data obtained in slices from wild-type mice in the presence of the A1R antagonist, DPCPX (50 nM) either in the absence (blue symbols and traces) or presence (pink symbols and traces) of AM251 (1 μM). **(C)** Quantification of LTP magnitude under the indicated conditions. In all cases LTP was induced by a strong-θ-burst. Data from WT mice in the absence of DPCPX (control WT, AM251 WT, panel **C**) are the same as that shown in **Figure 2B**, but is represented in this figure to allow comparisons with data from A_1_R KO mice and with data from WT slices in the presence of DPCPX. ^∗∗^*p* < 0.01; ^∗∗∗^*p* < 0.001 (*F*_(5,62)_ = 10.0, one-way ANOVA with Sidak’s correction). For further details see legend to **Figure 1**.

The magnitude of LTP induced by the strong-θ-burst in slices from A_1_R^-/-^ mice (65.5 ± 6.7%, *n* = 13) was not significantly different (*t* = 0.35, *p* > 0.05) from that obtained in A_1_R^+/+^ (68.1 ± 3.7%, *n* = 22, **Figure 5**). Remarkably, the CB_1_R inverse agonist AM251 inhibited LTP toward a similar value in both genotypes [A_1_R^+/+^: 29.6 ± 6.8%, *n* = 9; A_1_R^-/-^: 25.1 ± 9.3%, *n* = 8, *t* = 0.4, *p* > 0.05 when comparing genotypes; *t* = 4.1, *p* < 0.05 when assessing the effect of AM251 in A_1_R^-/-^ (control A_1_R^-/-^ vs. A_1_R^-/-^: AM251), **Figure 5**]. These data suggest that the inhibition of strong LTP caused by the inverse agonist of CB_1_R does not result from an enhanced A_1_R activation by released adenosine. To further confirm this, and to preclude any adaptation-like process due to genetic removal of A_1_R, we tested action of AM251 in A_1_R^+/+^ mice following inhibition of the A_1_R with DPCPX. Again, and in spite the presence of DPCPX at a concentration (50 nM) near 100 times its *K*_i_ for A_1_R (Bruns et al., 1987), thus expected to fully block A_1_R signaling, AM251 caused a marked inhibition of LTP induced by the strong-θ-burst (DPCPX: 68.0 ± 9.3%, *n* = 9; DPCPX+AM251: 32.5 ± 5.2%, *n* = 7, *t* = 3.3, *p* < 0.05, **Figure 5**).

Altogether the above data seem to indicate that the inhibition of LTP caused by preventing CB_1_R activation by eCBs is not due to enhanced activation of A_1_R by endogenous adenosine.

The absence of A_1_Rs also did not affect the inhibitory action of the CB_1_R agonist (500 nM WIN) upon LTP magnitude. Thus, in A_1_R^(-/-)^ mice the magnitude of LTP in the presence of 500 nM WIN (-0.57 ± 9.4, *n* = 5) was significantly lower (*t* = 4.1, *p* < 0.0001) than in the absence of WIN and not different (*t* = 0.5, *p* > 0.05) from LTP magnitude in slices from WT mice in the presence of 500 nM WIN.

### Astrocytes Do Not Contribute to the Enhancement of LTP Caused by eCBs

**FIGURE 6:**
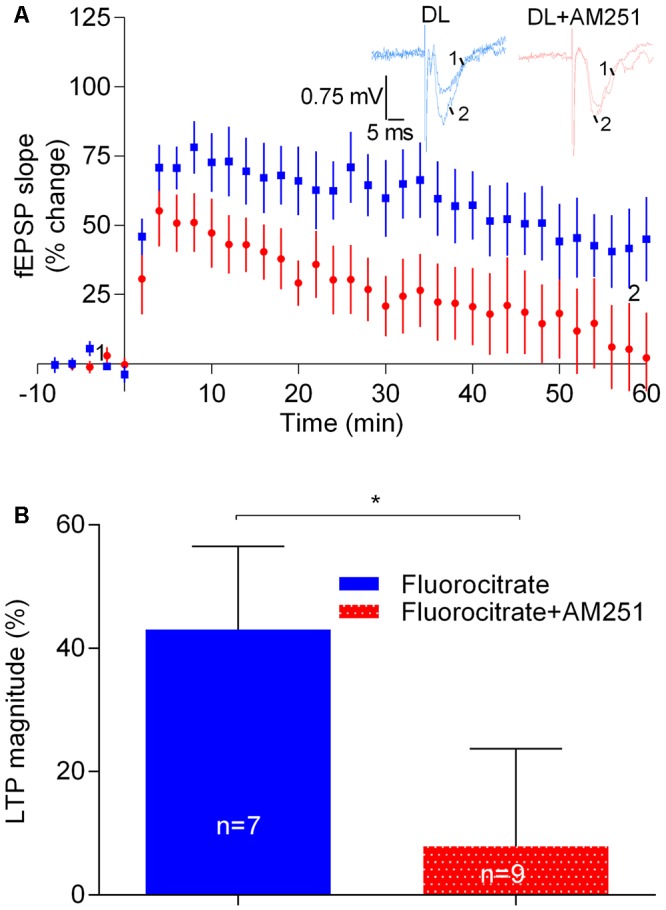
Astrocytes do not contribute to the enhancement of LTP caused by physiologically released eCBs. **(A)** Data obtained in slices treated with 200 μM Fluorocitrate to inhibit astrocyte metabolism, either in the absence (control) or in the presence of 1 μM of AM251. **(B)** Quantification of LTP magnitude under the indicated conditions. In all cases, LTP was induced by a strong-θ-burst. ^∗^*p* < 0.05 (*F*_(8,6)_ = 1.9, Student’s *t*-test). For further details see legend to **Figure 1**.

**FIGURE 7:**
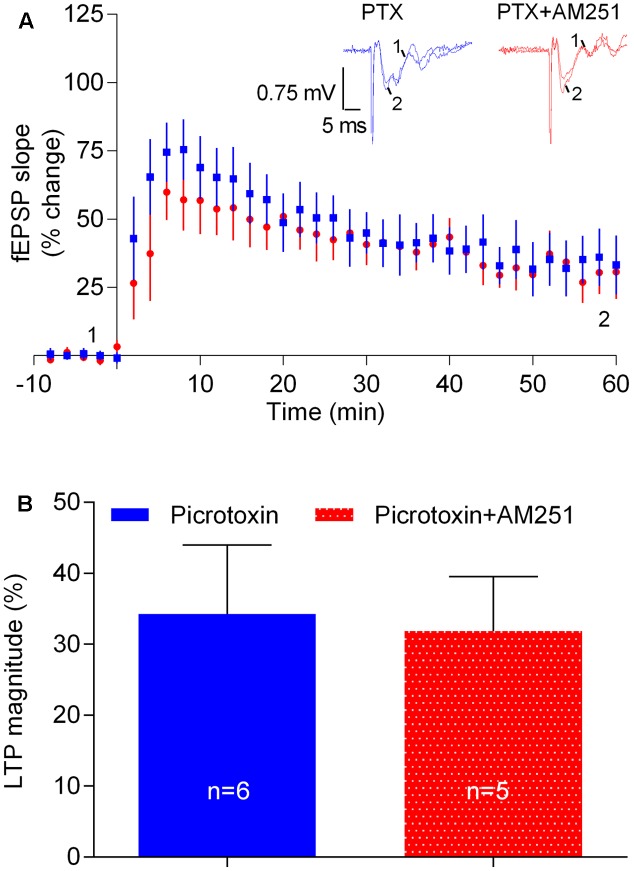
The influence of physiologically released eCB upon strong-θ-burst-induced LTP is lost upon blockade of GABAergic transmission. **(A)** Data obtained in slices treated with the GABA_A_ receptor antagonist, PTX (50 μM) in the absence (control) or in the presence of 1 μM of AM251. **(B)** Quantification of LTP magnitude under the indicated conditions. No significant differences between both conditions were found (*F*_(5,4)_ = 1.9, *p* > 0.05, Student’s *t*-test). For further details see legend to **Figure 1**.

Astrocytes are known to contribute to the facilitatory action of eCBs upon glutamatergic transmission (Navarrete and Araque, 2010). In addition, it is known that astrocytes, by releasing gliotransmitters, which then act in pre- and post-synaptic receptors, affect neuronal signaling and plasticity (Pascual et al., 2005; Henneberger et al., 2010). We thus hypothesized that the apparent facilitatory action of eCBs upon LTP induced by the strong-θ-burst would involve the astrocytes. To address that possibility we incubated the slices with the metabolic gliotoxin fluorocitrate (200 μM) for at least 20 min and allowed the fEPSP slopes to stabilize for at least 15 min before inducing LTP either in the presence or absence of AM251. As expected from previous reports (Bonansco et al., 2011) LTP magnitude was reduced in slices incubated with fluorocitrate (cf. data in **Figure 6** with **Figure 2**). Remarkably, however, under such conditions the CB_1_R inverse agonist, AM251, was still able to markedly inhibit LTP (fluorocitrate: 43.0 ± 13.5%, *n* = 7; fluorocitrate +AM251: 7.9 ± 15.8%, *n* = 9, *t* = 2.3, *p* < 0.05 vs. fluorocitrate alone; **Figure 7**).

These data suggest that the apparent facilitatory action of eCBs upon strong-θ-burst-induced LTP does not involve the astrocytes. This data also show that, at least under some experimental conditions, the strength of LTP induction may be even more determinant of the direction of the influence of eCBs upon LTP than the magnitude of LTP itself.

### The eCB-Mediated Enhancement of LTP Is GABAergic Transmission Dependent

Next we hypothesized that the apparent facilitatory action of eCBs upon LTP caused by the strong-θ-burst stimulation could be due to a preponderant inhibition of GABA release over glutamate release. To test this possibility, experiments were performed in the presence of the GABA_A_R antagonist, PTX (50 μM). LTP magnitude was smaller in the presence of PTX, as compared with its absence (cf. **Figures 2**, **7**), which may result from overactivity of glutamatergic transmission even before LTP induction. Remarkably, in slices in the presence of PTX, the inhibitory action of AM251 upon LTP was lost (PTX: 34.3 ± 9.7%, *n* = 6; PTX+AM251: 31.8 ± 7.7%, *n* = 5; 0.1, *p* > 0.05, **Figure 7**). The ability of PTX to prevent the inhibitory action of AM251 upon LTP should not be attributed to its ability to diminish LTP, since fluorocitrate also inhibited LTP and did not prevent the action of AM251 (cf. **Figures 6**, **7**).

The above data suggest that the apparent facilitatory action of eCBs upon LTP induced by the strong-θ-burst involves GABA_A_R-mediated GABAergic transmission, most probably resulting from eCB-induced inhibition of GABA release with consequent disinhibition of glutamatergic neurons.

### Prevention of CB_1_R Activation by eCBs Does Not Affect Basal Excitability

**FIGURE 8:**
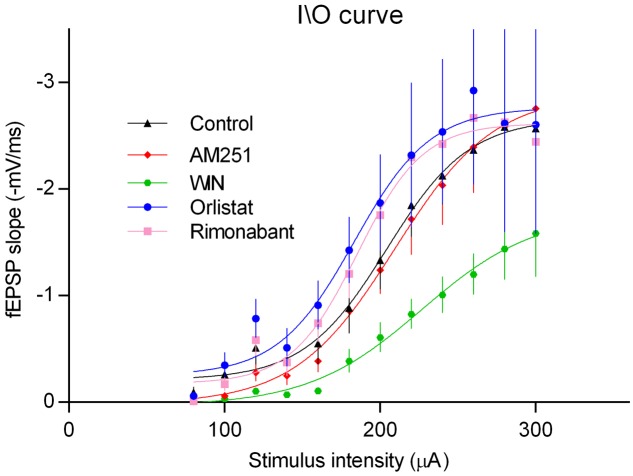
Input/output curves where fEPSP slopes values are plotted against the stimulation intensities (80 and 300 μA) in hippocampal slices under the drug conditions indicated. All values in the figure are represented as the mean ± SEM, *n* = (5–8). Statistical significance was assessed by two-way ANOVA followed by Sidak’s *post hoc* test, comparing multiple experimental groups; no significant differences among drug treatments were detected except for the WIN; ^∗^*p* < 0.05 as compared with the other drugs) (*F*_interaction(44,360)_ = 0.5, *F*_row factor(11,360)_ = 38.1, *F*_column factor(4,360)_ = 14.4, *t*-values: control/AM251 – 0.6, control/WIN – 5.2, control/Rimonabant – 1.1, control/Orlistat – 2.1).

Long-term potentiation can be influenced by changes in basal synaptic transmission. To evaluate if manipulation of 2-AG signaling could have a global influence upon excitability, input/output (I/O) curves were compared in the absence and presence of AM251, Rimonabant, or Orlistat. As illustrated in **Figure 8**, none of these drugs appreciably modified I/O curves compared with the control. Exogenous activation of CB_1_R using the CB_1_R agonist WIN, did however clearly altered the I/O curve, an action that can be attributed to its well-known ability to inhibit synaptic transmission at the CA1 area of the hippocampus (Serpa et al., 2009). The absence of influence of AM251, Rimonabant, or Orlistat in I/O curves but their influence upon LTP, indicates that transiently released 2-AG have a predominant influence over LTP rather than over basal synaptic transmission.

## Discussion

A main finding in the present work is that prevention of CB_1_R activation may affect CA1 LTP in an opposing way, depending on the strength of LTP induction and the magnitude of LTP itself. Thus, we show that a CB_1_R inverse agonist, a CB_1_R antagonist as well as an inhibitor of the formation of 2-AG, the main eCB in the hippocampus (Piyanova et al., 2015), leads to a facilitation of weakly induced LTP but to an inhibition of strongly induced LTP. This suggests that eCBs inhibit weak LTP while facilitating a more robust LTP. In accordance to the idea that physiologically released eCBs favor robust LTP is also the finding that an inhibitor of the degradation of 2-AG facilitates LTP induced by the strong-θ-burst. However, continuous activation of CB_1_R with an exogenous agonist or overproduction of a non-prevalent eCB, anandamide, leads to inhibition of strongly induced LTP. Overall, these findings are suggestive of dual actions of eCBs upon CA1 LTP depending both on the strength of LTP induction as well as on the nature of CB_1_R activators.

To our knowledge, this is the first time that dual action of cannabinoids upon hippocampal LTP is clearly shown. It is known for a long time that mice lacking CB_1_Rs have enhanced hippocampal LTP (Bohme et al., 1999; Jacob et al., 2012), compatible with the general idea of inhibitory actions of cannabinoids in the brain. Similarly, the work by Slanina et al. (2005) clearly showed that weak LTP, induced by a small number of pulses delivered at the CA1 area of hippocampal slices, is facilitated by CB_1_R blockade, also allowing the suggestion that eCBs inhibit weakly induced LTP. However, LTP induced by strong non-θ-burst high frequency stimulation (100 pulses for 1 s, or twice this paradigm separated by 20 s) was unaffected by CB_1_R blockade (Slanina et al., 2005), in clear contrast with the results herein reported for robust θ-burst-induced LTP. Species differences (mice in our study vs. rats in the study by Slanina et al., 2005) or age of the animals (adults in our study vs. adolescents in the study by Slanina et al., 2005) may account for these differences.

Facilitation of LTP by eCBs has been also reported, but again, those studies do not highlight a dual action of eCBs as a function of LTP magnitude. First evidence that eCBs facilitate CA1 hippocampal LTP was provided by the report that eCBs enable LTP induction by trains of EPSPs that are ineffective if eCBs are not allowed to act (Carlson et al., 2002). This action could be attributed to a eCB-mediated inhibition of GABAergic synapses (Carlson et al., 2002), and indeed it was later reported that upon removal of synaptic inhibition in a restricted area of the dendritic tree, there is a selective priming of nearby excitatory synapses by eCBs, which facilitate induction of CA1 hippocampal LTP (Chevaleyre and Castillo, 2004). Those studies allowed to understand the action of eCBs at the local circuitry and at the single neuron level, but do not inform on the global impact of eCBs upon LTP of pyramidal neurons. Using adult rats, De Oliveira Alvares et al. (2006) reported a marked inhibition of non-θ-burst high-frequency-induced CA1 LTP of fEPSPs by AM251, thus pointing that eCBs are required for robust LTP phenomena. More recently, Wang et al. (2016) reported that 2-AG and CB_1_R signaling is required for LTP of the lateral perforant path input to dentate gyrus neurons. In the study by Wang et al. (2016), however, robustly induced CA1 LTP was unaffected by preventing CB_1_R activation. In contrast, our data clearly point toward a facilitatory action of 2-AG and CB_1_R signaling on CA1 LTP induced by robust stimulation. Pattern of stimulation (θ-burst in both cases), or age (adult animals in both cases), cannot account for the differences. The difference may reside in the characteristics of the perfusion chamber, which may impact upon the accumulation of endogenous substances around the synapses. We used a slice submersion chamber, while Wang et al. (2016) used an interface chamber; submerging chambers likely favor the accumulation of endogenous substances. Lower level of oxygenation in submerged chambers may, in some studies, account for differences between data obtained in submerged or interface chambers (Hájos and Mody, 2009). However, this might not be the case since our chambers are provided with nylon mesh thus allowing oxygenation in both surfaces of the slice. Under our experimental conditions the oxygen pressure in the perfusion solution inside the chamber is 500–600 mmHg (Sebastiao et al., 2001). We used mice while Wang et al. (2016) used rats when testing the influence upon CA1 LTP, but species differences are unlikely to account for the dissimilarities since no marked differences were detected by Wang et al. (2016) while comparing LPP–LTP data in mice and rat hippocampal slices.

Pyramidal hippocampal neurons are under inhibitory control of GABAergic synapses, but also under control of several modulatory substances. Adenosine, an ubiquitous molecule released by neurons and glia, is able to modulate synaptic transmission and plasticity by operating high affinity G-protein-coupled receptors (Sebastião and Ribeiro, 2015). The adenosine A_1_R is highly expressed in the hippocampus and with a clear inhibitory action upon synaptic transmission and LTP (Dunwiddie and Masino, 2001; Boison, 2005; Sebastião and Ribeiro, 2015). The inhibitory action of CB_1_R upon GABA and glutamate release, as well as on synaptic transmission in the hippocampus are partially reduced by co-activation of A_1_R (Hoffman et al., 2010; Sousa et al., 2011), suggesting an interaction between these two modulatory pathways at the hippocampus (but see Serpa et al., 2009, 2015). The possibility that A_1_R-mediated attenuation of an inhibitory effect of eCBs could justify the apparent excitatory action of eCBs upon strongly induced LTP led us to test if the action of a CB_1_R blocker was affected by A_1_R deletion or A_1_R blockade. However, none of these significantly influenced the inhibitory action of AM251 upon strong LTP.

Astrocytes release several neuromodulatory substances, including purines (Henneberger et al., 2010; Lalo et al., 2014), and have been shown to contribute to the facilitatory action of eCBs upon hippocampal glutamatergic transmission (Navarrete and Araque, 2010). Metabolic inhibition of the astrocytes, a condition known to affect astrocytic signaling and release of gliotransmitters (Paulsen et al., 1987; Swanson and Graham, 1994; Bonansco et al., 2011) did, however, not affect the influence of AM251 upon LTP. This suggests that astrocytes do not play a major role in the facilitatory action of eCBs upon LTP.

A common conclusion in all studies reporting facilitation of LTP by eCBs is that it can be accounted by an influence upon GABAergic neurons (Carlson et al., 2002; Chevaleyre and Castillo, 2004; Wang et al., 2016). Accordingly, we also observed that the ability of the CB_1_R blocker, AM251, to inhibit LTP was lost in the presence of the GABA_A_R antagonist, PTX, thus reinforcing the conclusion that physiologically released eCBs facilitate LTP by restraining the inhibition of LTP imposed by GABAergic inputs. It has previously been shown that deletion of CB_1_R in GABAergic neurons leads to a decreased hippocampal CA1 LTP, whereas deletion of CB_1_R in glutamatergic neurons leads to enhanced LTP (Monory et al., 2015). It is therefore likely that the two stimulation conditions used in the present work lead to a differential influence of eCBs in GABAergic interneurons and glutamatergic neurons, so that under strong LTP induction conditions the influence of eCBs upon GABAergic neurons predominates.

CB_1_R are widely distributed in the central nervous system, mainly in the hippocampus, cortex, basal ganglia, and cerebellum (Marsicano and Lutz, 1999; Wilson and Nicoll, 2002). This receptor is localized in excitatory and inhibitory neurons (Katona et al., 2001; Wilson et al., 2001; Kawamura, 2006) and also in astrocytes (Hoffman et al., 2010; Han et al., 2012). Considering the neuronal compartment only, it has been estimated that about three quarters of all CB_1_R present in hippocampi are on GABAergic neurons while glutamatergic neurons contain about one quarter of all hippocampal CB_1_R (Steindel et al., 2013). Not all GABAergic hippocampal neurons express CB_1_R, these receptors being localized in cholecystokinin (CCK) positive neurons. CCK-positive neurons express higher levels of CB_1_R than the pyramidal cells (Marsicano and Lutz, 1999; Marsicano et al., 2003; Monory et al., 2006). It is thus not surprising that the apparent facilitatory action of eCBs upon strongly induced LTP results from an action upon GABAergic neurons, most probably by suppressing the inhibitory control exerted by CCK-positive basket cells over the pyramidal neurons.

Another relevant finding in the present work is the similarity between the effect of drugs that block CB_1_Rs or inhibit formation of 2-AG, the predominant eCB in the hippocampus (Piyanova et al., 2015), and the effect of sustained activation of CB_1_R by an exogenous agonist. It is worthwhile to note that the inhibitory action of Orlistat (2-AG synthesis inhibitor) and the inhibitory action of WIN (CB_1_R agonist) were both counteracted by the CB_1_R receptor blocker, AM251, clearly indicating that both are due to alterations in the level of CB_1_R activation. It is known for a long time that the CB_1_R activation inhibits LTP (Nowicky et al., 1987; Collins et al., 1995; Terranova et al., 1995; Stella et al., 1997; Paton et al., 1998; Basavarajappa et al., 2014), these inhibitory actions being usually interpreted on the light of the knowledge that exocannabinoids inhibit excitatory synaptic transmission. However, the novelty of the present work is the possibility to contrast, under the same experimental conditions, the action of drugs that continuously activate CB_1_R with those that reduce CB_1_R activation, allowing to suggest that CB_1_R can either facilitate or inhibit LTP as a function of several conditions, including the characteristics of CB_1_R activation, the strength of LTP induction, as well as the magnitude of LTP itself. Also worthwhile to note is the contrast, under the same experimental conditions, between the influence of a drug known to inhibit hydrolysis the predominant eCB at the hippocampus, 2-AG, which facilitates strong LTP in line with the idea of a facilitatory action of endogenous activation of eCBs, with that of drugs that unselectively prevent eCB metabolism or that prevent metabolism of anandamide only, both of which inhibit strong LTP. These findings highlight differences in the modulatory actions of the eCBs, which may be relevant to interpret some age-dependent differences in the neuromodulatory actions of cannabinoids. Indeed, the relative abundance of anandamide over 2-AG increases throughout age (Piyanova et al., 2015). Our data thus contribute to interpret apparently discrepant data, and strongly support the idea of a dual action of eCB signaling to sustain LTP.

## Conclusion

The data herein reported clearly show that manipulating eCB signaling may have opposing effects upon LTP, depending on the strength of LTP induction, inhibiting weak LTP and facilitating stronger LTP. This suggests that eCBs act as a high-pass filter, therefore likely reducing the signal-to-noise ratio of synaptic strengthening. Importantly, we also show that under the same LTP inducing conditions, prolonged activation of CB_1_R with exocannabinoids or blockade of CB_1_R may both impair LTP. Our data with drugs known to increase the accumulation of anandamide or 2-AG suggest that these two eCBs may differently affect LTP. Altogether, the data herein reported highlight a clear homeostatic control of eCBs and CB_1_Rs upon LTP. Disruption of this finely tuned homeostatic role of eCBs upon synaptic plasticity phenomena likely underlies the known deleterious influence of cannabinoid-based drugs upon memory.

## Ethics Statement

This study was carried out in accordance with the recommendations of “Directive 2010/63/EU.” The protocol was approved by the “iMM’s Institutional Animal Welfare Body – ORBEA-iMM and the National competent authority – DGAV (Direcção Geral de Alimentação e Veterinária).”

## Author Contributions

AS-C performed the experiments and quantified the data. AS and AS-C designed the experiments, analyzed, and discussed the data. MC provided breeding pairs for A1R knockout and WT mice. AS-C, MC, JR, and AS contributed to manuscript writing.

## Conflict of Interest Statement

The authors declare that the research was conducted in the absence of any commercial or financial relationships that could be construed as a potential conflict of interest.

## Supplementary Material

FIGURE S1 No appreciable differences between data obtained in males or females were detected. Values are represented as the mean ± SEM. The number of experiments in each condition is indicated in the bars. No significant differences between males and females were found, *F*_(11,102)_ = 7.2, *t*-values – Control WT:Control WT male = 0.9, Control WT:Control WT female = 0.5, AM251 WT:AM251 WT male = 0.9, AM251 WT:AM251 WT male = 0.6, Control KO:Control KO male = 0.06, Control KO:Control KO female = 0.05, AM251 KO:AM251 KO male = 0.1, AM251 KO:AM251 KO female = 0.07. For further details on the way to calculate LTP magnitude see the section “Materials and Methods” and legend to **Figure 1**.
