# Supplementary figures and images for: Dual Influence of Endocannabinoids on Long-Term Potentiation of Synaptic Transmission
Source: Front Pharmacol. 2017 Dec 19;8:921. doi: 10.3389/fphar.2017.00921 (PMC5742107; doi:10.3389/fphar.2017.00921)

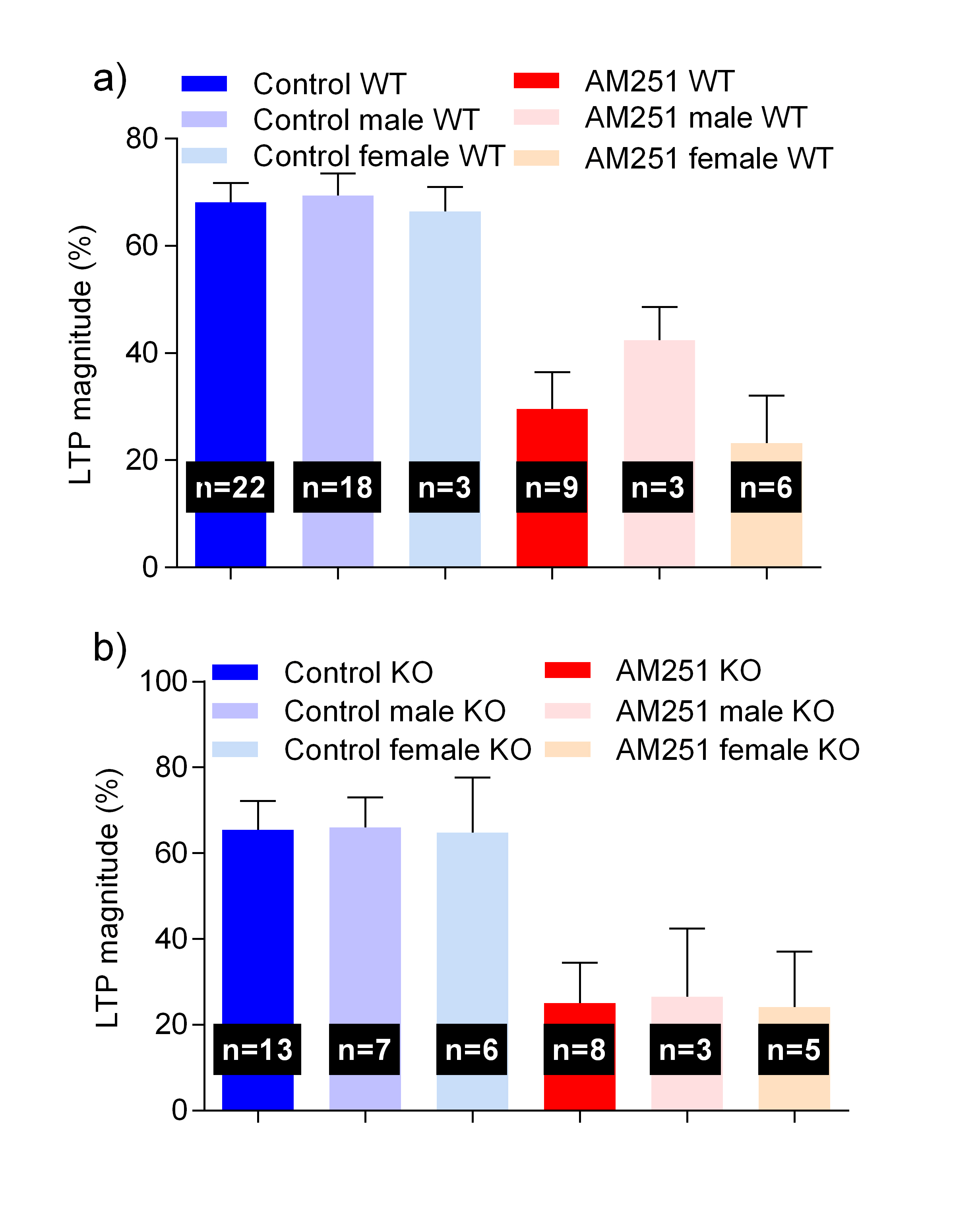

Supplement: FIGURE S1 — No appreciable differences between data obtained in males or females were detected. Values are represented as the mean ± SEM. The number of experiments in each condition is indicated in the bars. No significant differences between males and females were found, F(11,102) = 7.2, t-values – Control WT:Control WT male = 0.9, Control WT:Control WT female = 0.5, AM251 WT:AM251 WT male = 0.9, AM251 WT:AM251 WT male = 0.6, Control KO:Control KO male = 0.06, Control KO:Control KO female = 0.05, AM251 KO:AM251 KO male = 0.1, AM251 KO:AM251 KO female = 0.07. For further details on the way to calculate LTP magnitude see the section “Materials and Methods” and legend to Figure 1. [file Image_1.jpg]
